# Disinhibition of negative true self for identity reconstructions in cyberspace: Advancing self-discrepancy theory for virtual setting

**DOI:** 10.1371/journal.pone.0175623

**Published:** 2017-04-11

**Authors:** Chuan Hu, Sameer Kumar, Jiao Huang, Kurunathan Ratnavelu

**Affiliations:** 1 Institute of Graduate Studies, University of Malaya, Kuala Lumpur, Malaysia; 2 Asia-Europe Institute, University of Malaya, Kuala Lumpur, Malaysia; 3 Institute of Mathematical Sciences, University of Malaya, Kuala Lumpur, Malaysia; University of Texas at San Antonio, UNITED STATES

## Abstract

In face-to-face communications, to avoid sanctions and disapproval from others, people are more likely to hide negative aspects of their true self (such as socially undesirable personalities, minds, beliefs and consciousness) to avoid conflict with social norms and laws. The anonymity of cyberspace provides people a unique environment to behave more freely and openly with less restraint from the real word. Existing research related to online true self expression has mainly explored true self as an independent aspect of self. Regarding true self as a two-dimensional concept, this study investigates true self from the perspective of individuals’ self-guide and identity reconstruction in both online and offline world. Using qualitative research methods, the current study investigates 57 participants through interviews and questionnaires. Content analysis reveals four factors that motivate people to express more true self (especially negative true self) when reconstructing their online identity and involve true self as a part of their self-guide in anonymous environment. By incorporating true self as an important part of individuals' self-guide and identity online, the current study advances self-discrepancy theory, making it more comprehensive for cyberspace. The results are also interpreted based on self-determination theory. The theoretical contributions of this study are discussed and practical implications are also presented.

## Introduction

In the rapidly changing and developing world, identity is a significant analytic tool to understand society and human behaviors for researchers in different areas [1]. Generally, identity is defined as a certain “kind of people” that an individual expresses and be recognized by others in a given context [2,3]. Subjective to various given context, an individual’s identity is ambiguous and unstable [1,4]. Identity is also defined as the actual attributes that an individual presents in daily life, namely actual self [5]. Actual self was proposed by Higgins [6,7] in self-discrepancy theory, along with ideal self and ought self. While actual self reflects the current state of an individual, ideal self and ought self reflects an individual’s wishes and responsibilities, respectively. In the physical world, an individual’s self-guide, which consists of ideal self and ought self, serves as a significant standard for self-enhancement and self-improvement [8]. As a long accepted notion, people are motivated to positively enhance and improve themselves to achieve different goals in real world. For example, students need to study hard to enhance their own knowledge in order to get high scores in the examination. Professional athletes need to train hard to improve their ability to win matches. Consequently, individuals’ self-expression and self-guide in physical world are mostly positive, aiming to build a good image and obtaining positive evaluations from others [9].

However, the emergence of Internet changes the way people interact with others and constitutes a unique opportunity for the construction of individuals’ identity and self-guide [10–12]. By hiding the corporal body behind the screen, the way people express themselves has found a new dimension. Without the presence of physical appearances, individuals are able to express themselves more freely and openly online [13]. Moreover, the cyberspace provides people the chance to reconstruct their online identity based on their own discretions. People can hide and/or fabricate their personal information online, such as hiding their educational backgrounds or even faking their gender. With a reconstructed identity, people are able to express themselves online with less fear of sanctions and disapproval from others than in the physical world [5]. Thus, individuals’ self-expression online may no longer be as purely positive as it is in physical world. Additionally, the self-guide online (with which individuals are motivated to match their virtual identities) may no longer be limited to ideal and ought self. Individuals may also want to behave according to their “true self”, expressing intrinsic personalities, minds, beliefs and consciousness that are hidden in physical world.

### Literature review

True self was defined as the self that presents what people intrinsically think and believe [14]. Some existing literature related to true self online has focused on the relationships between personalities and the expression of true self. Marriott and Buchanan [15] proposed that the preference of true self expression of every single person is different and will be affected by personalities. McKenna et al. [16] found that social anxious people express their true self more easily online than offline. It was also suggested that introverted and neurotic people are more likely to take advantage of the anonymity online to express their true self [17]. While neuroticism and psychoticism were positively associated with the expression of true self on the Internet [18], conscientiousness was negatively associated with online true self expression [15]. Additionally, prior studies also indicated that expressing true self has some implications for relationship formation and maintenance in virtual settings [19]. Individuals who express more true self online are more likely to build new relationships with strangers and have “Internet only” friends [19]. People who behave in accordance with their true self are more likely to form close relationships with others on the Internet [16]. Moreover, some previous research also investigated the relationship between the expression of true self and the use of social network platforms. In Facebook, those who enjoy the freedom to express their true self have more self-oriented motivations for posting, and these postings reveal more personal and emotional content [4]. Moreover, people with high level expression of true self use Facebook more frequently for the purpose of new relationship establishment [20].

Even though much attention has been paid to the expression of true self online, existing research mainly explored true self as an independent aspect of the self. None of these studies regarded true self as a two-dimensional concept; neither did they investigate the expression of true self from the perspective of online identity reconstruction in anonymous environment. Drawing on self-discrepancy theory and self-determination theory, the current study aims to fulfill these gaps by proposing concepts of positive true self and negative true self to explore whether true self is a part of individuals’ identity and self-guides online, and the reasons why individuals choose to express more true self in anonymous environment.

The remainder of this paper is organized as follows. The next section presents the theoretical background. Then the research method is explained, followed by data analysis. After that, the results are further interpreted based on the theoretical background. Finally, the contributions of this study, its possible limitations, and future research are discussed.

## Theoretical background

### Self-discrepancy theory

Self-discrepancy theory proposed three domains of self: actual, ideal and ought self [6]. The actual self represents the characteristics that oneself or others think an individual possesses. It reflects the current state of an individual. The ideal self represents the characteristics that oneself or others wish an individual to possess ideally. It reflects someone’s hopes and aspirations. The ought self represents the characteristics that oneself or others believe an individual should or ought to possess. It reflects someone’s sense of duties, responsibilities and obligations [6,7].

An example could be used to better explain the difference between the three domains of the self. For a shy and lazy man who is currently an ordinary programmer in an IT company, the identity of programmer is the actual self that he expresses and be recognized by others in the company. His wish is to be a popular singer who is extrovert. The extrovert singer is the ideal self from his point of view. At the same time, himself and others (e.g., his wife) believe that he should work harder to better take care of the family. Then, the diligent worker and responsible husband is the ought self he should be.

The actual self composes an individual’s identity that presenting to others [21], ideal self and ought self are typically regarded as self-guide, which is the important standard for well-being and self-evaluation [22]. Different people may possess different self-guides: some people may use both ideal self and ought self as their self-guide, some people may only use ought self as self-guide, whereas some others may only use ideal self as self-guide [6]. However, it is likely that an individual’s identity (actual self) and self-guides (ideal self and ought self) are not consistent, in other words, different kinds of discrepancy may exist between the identity and self-guides. These discrepancies will lead to various psychological discomforts [6]. Greater discrepancy will induce greater psychological discomforts [23]. It is proposed in self-discrepancy theory that, in order to alleviate psychological discomfort, individuals are oriented to align their identity with the self-guide [6]. In other words, people are motivated to align their actual self in accordance with ideal and ought self to reduce the self-discrepancy between their identity and self-guide [6,7]. It is likely that the self-guide can be fulfilled more easily if an individual is able to reconstruct an online identity based on his/her own ideas.

Existing studies have investigated the self-discrepancy between individuals’ actual self (in physical world) and virtual self (in online world). It is found that, in avatar-based virtual environment (such as Massively Multiplayer Online Role-Playing Games), people’s offline personalities are associated with their actual-virtual self-discrepancy and the evaluations of virtual self [24]. The less discrepancy between actual self and virtual self is, the greater extent to which people experience the virtual identity as if it were their actual identity [25]. The actual-virtual discrepancy also mediates the relationship between social network site use and body image satisfaction [26]. In addition, it has significant influence on people’s psychological state, and in turn, affects the quantity and quality of their knowledge contribution in virtual communities [27]. However, most previous studies in related areas were using the traditional self-discrepancy theory and mainly focused on the effect of self-discrepancy. The current study aims to examine whether true self is a part of people’s identity and self-guide, trying to improve self-discrepancy theory and make it more comprehensive.

### True self

Rogers [28] suggested that true self is one of the significant aspects of an individual’s identity and people are highly motivated to express such important aspect in social interactions [29,30]. However, in face-to-face communications, if those aspects conflict with social norms and expectations, most people may choose to hide these attributes due to the fear of disapproval from others [20]. For example, McKenna and Bargh [31] mentioned that marginalized beliefs (such as ideological and homosexuals) can’t be fully expressed through communications and interactions in the real world.

Due to the constraints of social norms and laws in physical world, the personalities, minds, beliefs and consciousness that people express to others are mostly positive [9], as well as their self-guide. For example, students normally wish to have good academic performances throughout their study; children ought to be honest in daily life. However, the dark side of personality traits cannot be ignored. Many socially undesirable personality traits could be found in previous research about personality and social psychology, such as cynical, domineering [32], callousness, dishonesty, egocentricity [33], impulsivity, neuroticism, aggression [34], and so on. The expression of above mentioned undesirable personalities in physical world will conflict with social norms and expectations, hence, individuals may be negatively judged by others. As a consequence, in physical world, most people have to hide those intrinsic negative personality traits [35]. In other words, the negative aspect of personality does exist, but it is not suitable to be fully expressed in face-to-face communications in physical world [13]. Thus, an individual’s true self not only includes positive aspects, but also negative ones (namely positive true self and negative true self). As mentioned above, true self was defined as the self that people intrinsically think and believe [14]. Then, the positive true self includes an individual’s positive personalities, minds, beliefs and consciousness. The negative true self includes an individual’s negative personalities, minds, beliefs and consciousness. More specifically, the positive true self includes the positive aspects of true self that are in line with social norms and expectations in physical world. By contrast, the negative true self includes the negative aspects of true self that conflict with social norms and expectations.

Normally, an individual may try to hide the negative true self when presenting his/her identity in order to leave a better impression on others. For example, an employee needs to show assiduous to the boss even if s/he is a sluggard; most corrupt officials need to act rectitude and hide their avarice from others. However, the anonymity of the virtual setting enables people to express negative true self with less fear of negative social evaluation and disapproval by others [13,31]. As space transition theory proposed that, people behave differently when they move from one space to another [36]. For example, people may behave themselves in physical world due to their status and position. But when they move to cyberspace, they may act differently, such as expressing negative true self or even commit in cybercrimes [37–40].Without the limitation of corporal body, people can be partly or even totally anonymous in online world, which makes them feel less restrained by social norms and social sanctions. Additionally, in comparison to physical world, disclosing negative true self in anonymous online world may lead to less real costs (such as negative judgments from others, and damage to relationships) [13]. Thus, true self (especially negative true self) is more likely to be active when individuals reconstruct their identity in the virtual setting than in the physical world [13].

### Self-determination theory

Apart from self-discrepancy theory, self-determination theory, which also focuses on how human behaviors are motivated, is adopted in this study to better understand individuals’ needs of identity reconstruction in anonymous environment. Self-determination theory posits that people are motivated differently when they are doing something [41]. For example, an individual may be motivated to engage in an action because s/he wishes to do it or because s/he is pressured to do it. Human beings conduct their behaviors according to their own interests or the external benefits. This is also in line with the descriptions of self-guide in self-discrepancy theory [6,7]. Based on different reasons and goals, motivations that guide people’s behavior are distinguished into two types: intrinsic motivation and extrinsic motivation [42].

When intrinsically motivated, individuals do an activity naturally, following their inner interests. They are not doing it for separable consequences [43]. The participation and accomplishment of this activity fulfills their innate needs, like vanity. When extrinsically motivated, individuals engage in an action for separable outcomes rather than for intrinsic interest in the action itself [43]. In many cases, people take a certain action because it will bring them material rewards, like money.

More importantly, self-determination theory highlights three innate psychological needs, namely competence, relatedness and autonomy [42]. Competence refers to the need of feeling effective in actions and being able to express one’s capacities. The fulfillment of competence brings an individual the sense of confidence [42]. Relatedness refers to the need of being connected to others, especially to make friends with the people who share same inner interests. The fulfillment of relatedness brings an individual the sense of belongingness [42]. Autonomy refers to the need of acting according to one’s own volition and thought. When the need of autonomy is fulfilled, individuals regard their behavior as an expression of their sense of self [42].

Self-determination theory has been used to explain people’s behavior in social network platform use. It is suggested that individuals who experience a lack of intrinsic need satisfaction in offline life will try to compensate for these shortages through social network platform use [44]. Social anxious people tend to use social network platforms to fulfill their need for self-presentation [45]. Marketers are suggested to design precise strategies that meet people’s expectations and satisfy their basic psychological needs to improve their loyalty towards social network platforms [46]. The fulfillment of the three psychological needs is essential for individuals’ self-enhancement, self-improvement and psychological well-being [47]. Therefore, an individual’s behavior is motivated to fulfill the above mentioned three needs in both online and offline world [42]. In this study, self-determination theory is adopted to explain the reason why people are more likely to express more true self in the online world. The increased expression of true self in a reconstructed identity online may better fulfill people’s psychological needs than in physical world.

## Method

The current study aims to explore whether true self (including both positive and negative true self) will be involved as a part of individuals’ reconstructed identity and self-guide in anonymous cyberspace and the reason why individuals choose to express more true self online. Regarding to the ethical issues when exploring human behavior and consciousness, the whole research was approved by University of Malaya Research Ethics Committee (Document number: UM.TNC2/RC/H&E/UMREC-99). As good means to investigate and explore the complexity of human behavior and consciousness in naturalistic circumstances [48–50], qualitative research methods were adopted in this study. Strauss and Corbin [51] also proposed that qualitative research methods were suitable ways to understand and study social reality. Interview is one of the most important data collection methods, which is frequently used in qualitative research [52]. It keeps the subjects focused on the topic and allows the researcher to explore deeper when something interesting comes up [53]. It also helps researchers to explore subjects’ unique perspective or experience, allowing subjects to describe their thoughts in their own words [54]. Therefore, interviews were used in this study to explore participants’ ideas about true self.

### Research site

The research site of this study was QQ, a social network platform launched in China. By the end of March 2016, QQ had more than 800 million monthly active users in China [55]. It was adopted because of its high anonymous level. Unlike on some other social network platforms, such as Facebook on which users post so many “faces”, on QQ, users seldom reveal their real name or photo in profile. QQ users can create and join different communities based on their own interests to interact and communicate with others. For this kind of interest-based communities, many of them are very large (having more than one thousand members) and most members are geographically separated strangers. To protect privacy, some members will choose to reconstruct their online identities when interacting with a large amount of strangers in QQ community by hiding or even faking personal information in their online profiles, such as setting a carton picture as his or her profile photo and using an obvious pseudonym instead of revealing real name.

### Data collection

In this study, semi-structured interviews were adopted as the main method to collect data. However, since the expression of true self (especially the negative true self) is sensitive, some participants may feel inhibited to answer related questions in personal interviews. In this case, anonymous semi-structured questionnaires were also used as a supplementary data collection method. Open-ended questions, as well as closed-ended questions, were used in both interviews and anonymous questionnaires. With closed-ended questions, it is easier and quicker for participants to answer questions, by choosing from a list of ready-made options [56]. With open-ended questions, participants can provide answers in their own words freely and openly [57]. Then, it will be easier to investigate and explore an individual’s perception and consciousness related to true self [56,58,59].

To solicit participants, the interview invitations were sent out to three large interest-based QQ communities (each had more than one thousand members) which were recommended by QQ community search function under the category of interest. For the voluntary participants who are willing to take part in this study and attending our interview, they can choose two different ways to conduct interviews (through telephone or online chat) based on their needs. The purpose of this study and what kind of information would be collected and presented in the study were introduced to all the participants firstly. The interview began after the participants fully understood the research and agreed to continue. No official consent (written/oral) was provided, since all the data were collected and analyzed anonymously in this research. The data collection of this study was also approved by University of Malaya Research Ethics Committee (Document number: UM.TNC2/RC/H&E/UMREC-99). Each interview lasted approximately 25 minutes. During the interviews, some basic questions about participants’ personal information were asked as a warming up, including age, educational background and working fields. After that, the main interview questions were proposed to explore participants’ ideas about the expression of true self when they interact and communicate with others in both online and offline world. Additionally, thirty personality trait adjectives were selected from a published personality trait list [60]. Participants were asked to classify these personality trait adjectives into different categories based on their understanding in general, such as “suitable to express in real world”, “ideal self”, “negative true self”, and so on. The adjectives in the published personality trait list were rated based on people’s likeableness, from the “best” personality trait to the “worst” personality trait. The selection of the thirty personality trait adjectives was guided by the following criteria: a) the personality trait adjectives list is divided into three columns of equal length (top, middle, and bottom); ten words should be selected from each column; b) the translation into Chinese of a personality trait adjective to be selected should be straightforward; c) the personality trait adjective to be selected should be easy to understand and fits the research topic of this study. The interviews were conducted in Chinese (see S1 and S2 Files), given that all the participants were from Mainland China, The collected data were translated into English by one researcher and double-checked by other two researchers.

### Participants

**Fig 1:**
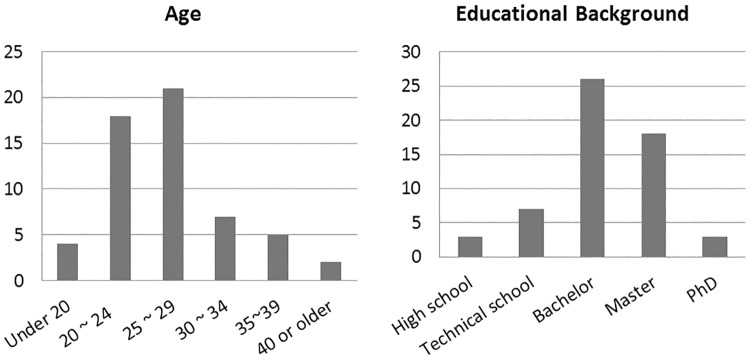
Participants’ demographic information.

In total, 57 participants were involved in this study (including 27 males and 30 females) and all of them are from China. These participants had different educational backgrounds, varying from high school graduates to PhDs. And they came from more than ten different fields such as computer science, management, manufacturing and so on. In addition, the age of these participants ranged from under 20 years old to over 40 years old (see Fig 1). The average time that all the participants spent online everyday was 5.35 hours.

## Data analysis and results

As mentioned above, the interview included both closed-ended and open-ended questions. For the data collected from the closed-ended questions, the results were easily quantifiable [61–63]. Because participants were required to select answers from pre-selected options, unique or unanticipated answers are not allowed in this kind of questions. For the data collected from the open-ended questions, content analysis method was adopted to analysis the data. Content analysis is a widely used method in qualitative research. It is flexible for analyzing text data obtained from interviews and survey questions [64]. The main feature of content analysis is to classify large amount of text into smaller number of categories [65], aiming to generate a condensed description of the phenomenon [66]. In this study, content analysis was used to generalize the reasons why people prefer to express more true self in online world than in the physical world. The unit of analysis was the thematic unit. The words, phrases and sentences expressing similar ideas were coded as a unit. The following are two examples of coding.

**Fig 2:**
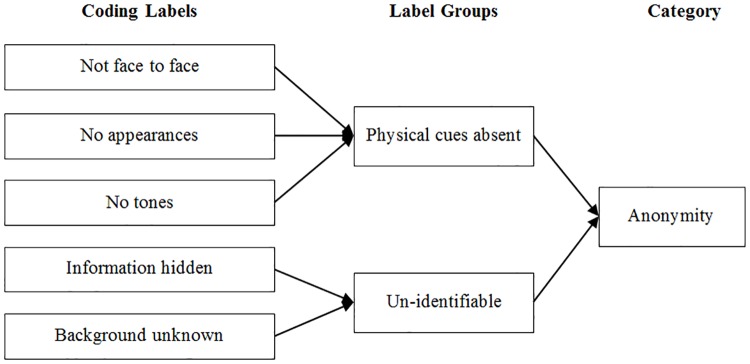
An example of category generation.

The first example is: “I can hide a lot of information, like names, addresses.” This sentence was labeled as “information hidden”. This label reflects the situation in which people hide their personal information on purpose, making them anonymous intentionally. It represents subjective choices. The label “information hidden” focuses on basic personal information (such as name and gender), while another similar label “background unknown” focuses on more confidential information related to background (such as family background and financial background). The second example is: “It’s not face to face. I don’t have to meet anyone directly.” “I won’t be affected by face to face factors” These two sentences were both labeled as “not face to face”, because both sentences expressed the idea that the communication online is not face to face. Even though people usually don’t meet each other face to face in online interactions, the first example was not labeled as “not face to face”. Because in this study, the label “not face to face” emphasizes on the fact that physical cues are absent. It reflects an objective status of online interactions, just as the situations shown in the second example. The data was coded iteratively until no more new labels emerged. Then, all the coding labels were compared with each other, and similar ones were placed into the same label group. In the end, label groups that are interrelated were classified into high-level categories. Fig 2 is an example of how the categories were generated from coding labels.

### Expression of true self

The majority of the participants (96.49%) agreed that everyone has both positive and negative personalities, thoughts, and beliefs. They mentioned various negative personality traits that people may have, such as violent, selfish, mean, falsity, aggressive, lazy, and so on. Those negative personality traits are part of true self, but are not socially desirable [32]. The expression of those traits in the real world may cause negative social evaluation and disapproval from others. Therefore, they cannot be fully expressed in face-to-face communications.

However, due to the high anonymous level of QQ, the cost of expressing above mentioned social undesirable personalities in QQ communities is much less than in the physical world. Thus, QQ community members can express their negative personalities, minds, beliefs and consciousness to greater extent with less concern. So the current study asked participants to distinguish whether the selected thirty personality trait adjectives [60] are suitable to be expressed in physical world, given that all the personality traits (both positive and negative ones) could be expressed in anonymous environment if they want. Additionally, to explore how individuals’ self-guides are constructed in both online and offline world, participants were also asked to classify the selected personality traits into the four domains of the self based on their own understanding in general. The results are shown in Table 1.

**Table 1:** Summary of results.

Categories	Personality Traits	Ideal Self	Ought Self	Positive True Self	Negative True Self
**Unambiguously positive personalities**	Honest	**26.32%**	**33.33%**	**40.35%**	0.00%
	Obedient	**33.33%**	**36.84%**	**29.82%**	0.00%
	Thrifty	**22.81%**	**40.35%**	**36.84%**	0.00%
	Righteous	**31.58%**	**38.60%**	**28.07%**	1.75%
	Cautious	**33.33%**	**29.82%**	**36.84%**	0.00%
	Responsible	**22.81%**	**38.60%**	**38.60%**	0.00%
	Polite	**33.33%**	**35.09%**	**31.58%**	0.00%
	Disciplined	**24.56%**	**45.61%**	**29.82%**	0.00%
	Objective	**12.28%**	**57.89%**	**29.82%**	0.00%
	Materialistic	**14.04%**	**49.12%**	**33.33%**	3.51%
	Patient	**28.07%**	**49.12%**	**22.81%**	0.00%
	Frank	**26.32%**	**26.32%**	**47.37%**	0.00%
	Modest	**33.33%**	**36.84%**	**29.82%**	0.00%
	Persistent	**12.28%**	**47.37%**	**28.07%**	12.28%
	Independent	**26.32%**	**40.35%**	**33.33%**	0.00%
	Humorous	**17.54%**	**40.35%**	**40.35%**	1.75%
	Self-confident	**26.32%**	**45.61%**	**26.32%**	1.75%
	Generous	**33.33%**	**35.09%**	**31.58%**	0.00%
**Slightly negative personalities**	Daydreamer	17.54%	5.26%	24.56%	52.63%
	Impulsive	3.51%	3.51%	12.28%	**80.70%**
	Vain	3.51%	1.75%	7.02%	**87.72%**
	Conceited	5.26%	1.75%	10.53%	**82.46%**
	Neurotic	3.51%	0.00%	8.77%	**87.72%**
**Unequivocally negative personalities**	Superficial	0.00%	1.75%	7.02%	**91.23%**
	Rude	1.75%	5.26%	3.51%	**89.47%**
	Greedy	3.51%	5.26%	3.51%	**87.72%**
	Domineering	0.00%	7.02%	1.75%	**91.23%**
	Cynical	1.75%	8.77%	0.00%	**89.47%**
	Uncivil	1.75%	3.51%	3.51%	**91.23%**
	Antisocial	1.75%	5.26%	3.51%	**89.47%**

As shown in Table 1, the thirty personality trait adjectives fell into three categories (unambiguously positive personalities, slightly negative personalities and unequivocally negative personalities). The first category includes eighteen personality trait adjectives. More than 80% of the participants thought these personality traits are suitable to be expressed in physical world. All of these eighteen personality traits turn out to be unambiguously positive personalities which are in line with social norms and laws in the physical world. The second category includes five personality traits. Whether these traits are suitable to be expressed in real world is controversial. Approximately half of participants thought these personality traits cannot be expressed in real world, while the other half of participants thought it is suitable to express these traits in physical world. These five personality traits are slightly negative personalities; hence, some people may think the expression of these personalities will not cause huge damage to their life. In some extremely cases, these slightly negative personality traits may even be helpful in their daily life. The third category includes seven personality trait adjectives. Less than 20% of the participants thought these traits are suitable to be expressed in the real world. The seven personality traits are unequivocally negative which conflict with social norms and laws in the real world.

The results are consistent with Anderson’s [60] research about personality traits, which ranked 555 personality traits from the “best” to the “worst” based on people’s likeableness. The eighteen personality trait adjectives in the first category distributed in the top and middle of Anderson’s [60] personality trait list, indicating that most people hold positive or neutral attitude towards them. The five personality trait adjectives in the second category located in the middle and bottom of the personality trait list, indicating that most people hold neutral or negative attitude towards them. The seven personality trait adjectives in the third category located in the bottom of Anderson’s [60] personality trait list, indicating that most people hold negative attitude towards them.

Taking the above mentioned results into account, participants’ responses about which domain of the self these personality traits belong to are also meaningful. For the first eighteen unambiguously positive personality traits that definitely can be expressed in physical world, participants prefer to regard them as ideal self, ought self and positive true self when (re)constructing identity and self-guide. This suggests that people’s identity and self-guide in physical world are mostly positive and they believe that positive true self is suitable to be expressed in physical world. For the seven unequivocally negative personalities that definitely cannot be expressed in physical world and four slightly negative personalities (except daydreamer) that can be expressed in real world in some extremely cases, participants tend to treat them as negative true self when (re)constructing identity and self-guide. This suggests that people think negative true self is not suitable to be expressed in physical world. The results also indicate that negative true self is totally different from positive true self, therefore, it is reasonable to propose true self as a two-dimensional concept. A possible explanation for the exception of daydreamer could be that daydreams about significant others are associated with the feeling of happiness, love and connection [67]. People can obtain some positive experience from daydreams. Therefore, some people may not regard daydreaming as negative true self.

### Motivations for true self expression online

47 participants (82.46%) agreed that individuals can express their true self more freely and openly online than offline. The majority of them also admitted that they would like to follow their true self when they are online. Content analysis was used to analyze the reasons why people express more true self online. Eventually, four factors were summarized, namely anonymity, less restraints, online-offline dissociation, and online listeners.

Thirty-two participants mentioned that anonymity is one of the motivators for expressing true self online. Since the communications online are mostly text-based, there is no face to face contact. Just like one participant indicated: “It’s not face to face. I don’t have to meet anyone directly.” People are no longer inhibited by physical cues (e.g. disapproval signs from others) [68]. They can stay behind the screen, partly or even totally hide their real personal information and background, and express whatever they want. For example, a participant mentioned that: “On the Internet, you don’t have to face anyone. Nobody knows what you look like, who you really are. This anonymous environment makes me feel safe. I can express myself as freely as I want.” Additionally, due to the anonymity online, it is difficult to make someone responsible for what s/he does. For instance, one of the respondents stated: “I barely need to be responsible for my behavior online.” In cyberspace, individuals are able to hide and fabricate their personal information when reconstructing identity [69]. Even if a person does some deviant actions (e.g. behave according to negative true self), s/he can avert responsibility for those behaviors in anonymous environment [68]. Just as mentioned by another respondent: “They (other Internet users) can’t find me. I can get disappeared whenever I want.”

Twenty-six participants indicated that they would like to behave according to true self online because the restraints online are much less than offline. There are various constraints in real world. Individuals need to obey different rules, try to meet social expectations, and maintain various relationships. Just like one participant indicated: “In real world, sometimes I am pressured to hold back my real thoughts.” People usually have to behave themselves, and can’t express their true self freely [13], especially when the intrinsic personalities, minds, beliefs and consciousness are not socially desirable (such as negative true self). But the situation is different in the anonymous virtual world. There are much less laws, rules and norms online. People can behave more freely, as if the legal and moral restrictions are temporarily suspended [68]. They have the freedom to reconstruct their virtual identity and behave according to their true self in anonymous environment. For instance, one of the participants mentioned: “There is less restraints on the Internet, so there is not much to worry about. It is OK to express my true self freely and openly, especially some negative aspects.”

Sixteen participants stated that they express more true self in their online identity and regard true self as online self-guide because virtual setting and physical world are not directly connected. The dissociation of online world and real world enables people to behave with less concern. People may feel less vulnerable about acting out online when they are able to separate their online behaviors from their offline identity and real life [68]. Even if individuals follow their negative true self, doing things they usually won’t do, their actions online will not be linked to their real life directly. As indicated by one participant: “It’s just like wearing a persona. My online image won’t affect my offline image. I won’t get myself in trouble when I express my negative true self online.”

Ten participants regarded other Internet users as good listeners for what they really think and believe. For example, one participant stated: “It is easier to tell what I really think to strangers online. I don’t have to worry about the consequences of expressing my true self.” This kind of interaction with strangers online is similar to the case of “strangers on a train” [70], in which people open up and talk to the stranger sitting in the next seat. They may talk about intimate details that they have never told to their colleagues or even friends and family. Additionally, some participants are looking for support from others. Just as mentioned by one of the participants: “On the Internet, you can always find a group of people who share the same idea with you. They understand you, and won’t judge you negatively. Expressing my true self to these people makes me feel supported.”

## Discussion

### Advancing self-discrepancy theory with true self

Traditional self-discrepancy theory proposed three domains of self: actual self, ought self and ideal self. And it also suggested that ought self and ideal self are significant standards and directions for people to form and present their identity [6,7]. However, we found that, in addition to ought self and ideal self, people are also willing to behave in accordance with their true self in anonymous online world. The traditional self-discrepancy theory is not comprehensive enough in interpreting the reconstruction of identity and self-guide, especially in anonymous environment. Therefore, by incorporating true self, the current study proposed an advanced self-discrepancy theory in which true self is also an important part of individuals’ identity and self-guide. The results of this study validated the advanced self-discrepancy theory and provided possible explanations of why true self was left out in the traditional self-discrepancy theory [6,7].

The results revealed that unambiguously positive personality traits were classified into not only ought self and ideal self, but also positive true self. By definition, positive true self is the intrinsic ideas about what people really think and believe, the ought self and ideal self in self-guide consist of individuals’ own ideas about their duties, wishes and aspirations. In some cases, the duties, wishes and aspirations are also the ideas that people intrinsically wish to achieve. So it is difficult to distinguish positive true self from ought self and ideal self. In other words, the positive true self is generally overlapping with the ought self and ideal self. This is also in line with our results that all the unambiguously positive personalities were evenly distributed in the three domains of the self (ideal self, ought self and positive true self). This might be the reason why true self was not included as the self-guide in traditional self-discrepancy theory [6,7].

Additionally, the exclusion of true self in traditional self-discrepancy theory may also be explained from the perspective of self-determination theory. Self-determination theory suggests that external regulations (extrinsic motivations) could be transformed to internal regulations (intrinsic motivations) [71]. This process is referred to as internalization [72]. There are two different types of internalization: introjection and integration [71]. In introjection, an external regulation is partially internalized, which means an individual “takes in” the regulation, but doesn’t accept it as his/her own [47]. This individual does something because s/he feels that s/he has to do it, not because s/he wants to do it, such as the duties, responsibilities and obligations in ought self. In integration, an external regulation is fully internalized, which means an individual recognizes the value of an activity and fully accept and integrate it with his/her own goals [47], such as the intrinsic beliefs, consciousness hopes and aspirations in positive true self and ideal self. However, it is difficult for an individual to distinguish whether an external regulation is partially or fully internalized. That is the reason why people cannot distinguish positive true self from ought self and ideal self clearly, which is also in line with our result that positive true self is mixed up with ought self and ideal self in physical world. This might be another explanation of why true self was left out in traditional self-discrepancy theory [6,7].

**Fig 3:**
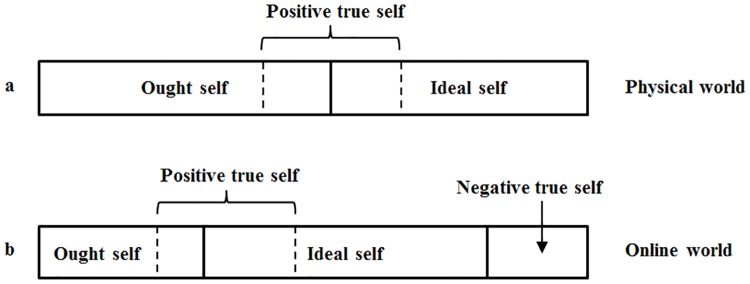
Self-guide in physical world and online world.

Even though there is no clear distinction between positive true self and ought self or ideal self, positive true self is an important part of their identity and self-guide [28]. People are highly motivated to express such essential aspect in social interactions [29,30]. The results also revealed that, negative true self is not suitable to be expressed in physical world. Thus, an individual’s self-guide in the physical world consists of ought self, ideal self and positive true self (see Fig 3a).

The results of this study also indicated that, people may reconstruct their identity in order to express more true self (especially negative true self) in anonymous online environment. As the results revealed, people are pressured by various constraints in the real world. True self, especially negative true self, cannot be easily expressed in daily life. It was suggested that individuals’ true self is more likely to be active in the virtual setting than in the real world [13]. This is also reflected in the results about the allocation of negative personalities in people’s identity and self-guide. The personality trait adjectives which were classified into negative true self by participants with high percentage were also classified into the category that are not suitable to be expressed in the physical world or can be expressed in real world in some extreme cases. Compared to the physical world, most people regard the virtual setting as anonymous, free and open environment, which provides a better platform to act out true self (especially negative true self). In the virtual setting, individuals are able to express more negative true self, when reconstructing their identity and self-guide. Therefore, apart from ought self, ideal self and positive true self, negative true self also composes an important part of an individual’s self-guide in the online world (see Fig 3b).

By expressing more true self with a reconstructed virtual identity in online world, an individual are able to reduce the discrepancy between his/her identity and self-guide, therefore, become motivated and satisfied [6]. People are intrinsically motivated to regard true self as their self-guide and include more true self when reconstructing their identity in cyberspace. The expression of true self online also fulfills individuals’ needs of competence, relatedness and autonomy, and it is essential for individuals’ psychological growth, integrity and well-being.

### Self-determination theory

The current study also found four factors that motivate people to express more true self in anonymous environment. According to self-determination theory [42], individuals’ behaviors are motivated to fulfill three innate psychological needs. Behaving according to more true self online fulfills individuals’ need of competence, relatedness and autonomy.

The need for competence suggests that people desire to perform effectively, to obtain the experience of being competent [47,73]. The results of this study reveal that the online-offline dissociation diminishes people’s concerns on acting out both positive true self and negative true self online. Their behaviors online won’t be directly connected to their physical life. The anonymity and less restraint online also enable individuals to reconstruct their virtual identity more freely and openly, including express more true self. Therefore, people can behave in accordance with their own ideas more effectively during communications on the Internet than face-to-face, making them feel competent. For example, a shy person, who feels frustrated when interacting with strangers in real world, may be competent in online communications without the disclosure of corporal body. These results are in line with previous studies which suggest that technological communication is a facilitator for people who can’t articulate their inner thoughts and/or feelings effectively in face-to-face communications due to the risk of embarrassment or disapproval [74,75]. McKenna et al. [16] also found that social anxious people can better express their true self on the Internet than in real world. Additionally, introverted and neurotic people who have difficulties in face-to-face social interactions are more likely to benefit from the anonymity of the Internet and express their true selves in cyberspace [17].

The need for relatedness implies that people desire to be connected to, and be supported by others [47,76]. Individuals want to be cared for by others, and want to be part of a community [42]. For the people who regard other Internet uses as listeners, they are actually hoping that their true self is heard, understood and/or accepted by others. The result shows that people feel being understood and supported when they reveal their true self to like-minded people online. Disclosing true self would create empathic bonds between people and facilitate the establishment of close relationships [13]. Therefore, acting out true self fulfills individuals’ need of relatedness. Individuals who express more true self online are more likely to build new relationships with strangers and have “Internet only” friends [19]. Previous research found that people are more likely to establish close relationship with others in the virtual setting with greater extent expression of true self [16]. And this kind of online relationship is under control. People can build or break these relationships based on their own volition and thought.

The need for autonomy suggests that people desire to act with a sense of volition in order to feel psychologically free [47]. Our findings indicated that the various constraints in real world would prevent people from expressing their true self. In the physical world, normally, it is difficult to lay bare one’s own bosom with others, especially negative ones, because no one is willing to share his/her dark aspects of the self to his/her friends and family. But people have the need to express those personalities, minds, beliefs and consciousness [28]. The opportunity to express the greater extent of true self (especially negative true self) online satisfies individuals’ need for autonomy. Their behaviors of acting out true self online are self-determined. Based on the results, it is clear that there is less restraint and less responsibility on the Internet, people can decide what to say and what to do based on their own ideas. They can be completely anonymous, and behave according to their true self without fear of being identified and negatively evaluated. This is supported by prior study, which suggested that autonomy in anonymity enables individuals to experiment with new behaviors and explore their hidden identity [77].

## Conclusion

Bargh et al. [13] suggested that true self actually existed psychologically, but is not fully expressed in social life. Due to the above mentioned four factors (anonymity, less restraints, online-offline dissociation, and online listeners), most people agree that Internet provides a more open and freer environment for self-expression, especially for negative true self. The current study found that true self is an important part of an individual’s identity and self-guide. In order to make self-discrepancy theory more comprehensive in interpreting people’s identity and self-guide, this study incorporated true self into the theory as the fourth domain of the self, proposing an advanced self-discrepancy theory. We found that positive true self overlaps with ought self and ideal self when (re)constructing people’s self-guide and identity in both online and offline worlds. The negative true self is hidden to avoid conflictions with social norms and expectations in physical world. When in anonymous online environment, the negative true self which is hidden in physical world will be discharged due to the release of social norms and laws. Therefore, individuals’ self-guide in physical world consists of ideal self, ought self and positive true self, while their self-guide in online world consists of ideal self, ought self, positive true self and negative true self. The expression of negative personalities, minds, beliefs and consciousness is one of the most significant distinctions for true self expression in physical world and online world. When reconstructing identity in cyberspace, individuals are eager to align their identity with self-guide. The self-discrepancy between their virtual identity and self-guide will decrease because they are able to express more true self (especially negative true self) with a reconstructed identity online. As a consequence, people will be more motivated and satisfied in anonymous online environment [6,7]. Meanwhile, the expression of more true self online also satisfies the three intrinsic needs proposed in self-determination theory [42,47], making people more motivated online.

## Contributions

The present study contributes to related literature in several ways. First, this study regards true self as a two-dimension concept (positive true self and negative true self) to investigate the differences of true self expression in both physical world and online world. To the best of our knowledge, this is the first time that true self is delved into from two different perspectives, which may facilitate the research about human behavior in cyberspace, such as Internet fraud and cybercrime. Most importantly, this study advanced self-discrepancy theory by incorporating true self (including both positive and negative true self) as an important aspect of an individual’s identity and self-guide, suggesting that, as self-guide, the positive true self is overlapping with ought self and ideal self, and can be expressed in both online and offline world; whereas negative true self, which conflicts with social norms and expectations, is not suitable to be expressed physical world. Additionally, it is found that expressing true self online is a self-determined behavior. The current study also found four factors that motivate people to express more true self in anonymous environment. Individuals’ needs of competence, relatedness and autonomy are fulfilled when they behave according to their true self online.

The findings of this study also have some practical implications. The online service providers are advised to implement features that support virtual identity reconstruction, so that individuals can express more true self with reconstructed identities. However, if the service provider aims to build a positive and harmonious environment, some monitoring mechanisms should be implemented at the same time. Otherwise, some people may express their negative true self excessively, such as spreading a lot of information about violence.

## Future research and limitations

In addition to the theoretical and practical contributions mentioned above, the results of this study also create some new directions for future research. The main focus of the current study is true self, suggesting that true self may be more apparent in anonymous online environment. The excessive expression of negative true self may have negative influences on other Internet users (such as cybercrime) [78]. Future research could be conducted to explore the association between the negative true self expression online and the consequences it may cause. In addition to the expression of true self, previous research pointed out that people tend to engage in “impression management” and express their ideal self to build a better public image on less anonymous social network platforms (such as Facebook) [79]. Future research could be directed to investigate whether the expression of ideal self is more apparent in more anonymous online environment. Additionally, due to online-offline dissociation, people may feel less inhibited by social norms in online world. They may no longer follow some of the social norms they used to follow in real life (such as be honest to others). Therefore, it is likely that there might be a decrease in the duties and responsibilities that people want to take in online world. Their ought-self guide online might decrease when compared with that in offline. Moreover, people are able to reconstruct their online identity based on their own ideas [5]. It is possible that people might have more wishes online than offline, because it would be easier to fulfill their dreams and wishes online through identity reconstruction. The dreams that are not possible in real life may come true in online world. Future research could be conducted to explore whether individuals’ ought-self guide and ideal-self guide in online environment differ from those in real life.

Even though this study has valuable contributions, it also has limitations. The participants of this study were recruited from interest-based QQ communities in Mainland China. The research is done in a given context. The generalizability of the findings may be limited by the specific context. Future research could be conducted in other contexts to validate the results. Cross-cultural research between China and some other countries might be a good choice to investigate whether political and cultural factors have influence on people’s expression of true self. Additionally, Anderson’s personality trait list includes 555 adjectives. To reduce the complexity of the interview, this study only selected 30 personality trait adjectives from the list to explore whether true self (including both positive and negative true self) will be involved when an individual reconstructs his/her self-guide and identity in cyberspace. More personality trait adjectives could be used in future research.

## Supporting information

S1 File Questionnaire in Chinese. (DOCX)

S2 File Questionnaire in English. (DOCX)
