# Supplementary material for: Disinhibition of negative true self for identity reconstructions in cyberspace: Advancing self-discrepancy theory for virtual setting
Source: PLoS One. 2017 Apr 11;12(4):e0175623. doi: 10.1371/journal.pone.0175623 (PMC5388501; doi:10.1371/journal.pone.0175623)
Supplement: S1 File — (DOCX) [file pone.0175623.s001.docx]

**问卷调查：网络世界中的“真实自我”**

互联网的出现，改变了人与人之间的交流方式，给人们创造了一个独特的展示自我的机会，让人们可以表达隐藏在内心深处的想法、意识以及个性。在匿名的网络世界中，人们可以尽情地展现“真实自我”，而不用担心受到处罚或被他人否定。**“真实自我”被定义为人们内心深处的真实性格、思想和信念。**本项研究致力于探索人们在互联网与现实生活中自我表现的差异，特别是在表现“真实自我”时的差异。

      非常感谢您的参与！本次问卷调查是**完全自愿且匿名**的，所有收集的数据将只用于学术研究，并只发布经过统计的结果。

1. **您是否同意人们的性格、思想和信念，既有积极的一面又有消极的一面？**
2. **与现实世界相比，您是否认为在网络世界中您可以更自由地表达“真实自我”？为什么？**
3. **通常情况下，以下性格是否适合在现实世界中展示？**

|  | **是** | **否** |
| --- | --- | --- |
| 诚实 |  |  |
| 做白日梦 |  |  |
| 谨慎 |  |  |
| 幽默 |  |  |
| 冲动 |  |  |
| 嚣张跋扈 |  |  |
| 有责任心 |  |  |
| 客观公正 |  |  |
| 贪婪 |  |  |
| 现实 |  |  |
| 肤浅 |  |  |
| 有礼貌 |  |  |
| 有恒心 |  |  |
| 孝顺 |  |  |
| 虚荣 |  |  |
| 有耐心 |  |  |
| 粗鲁 |  |  |
| 神经质 |  |  |
| 慷慨 |  |  |
| 自律 |  |  |
| 愤世嫉俗 |  |  |
| 独立 |  |  |
| 反社会 |  |  |
| 直率 |  |  |
| 谦虚 |  |  |
| 自信 |  |  |
| 自负 |  |  |
| 节俭 |  |  |
| 不文明 |  |  |
| 正直 |  |  |

1. **除了上述列举的性格，请您再列举两到三个只适合在网络世界中表现，而不适合在现实世界中表现的性格。**
2. **请根据定义及您的理解，为以下词汇选择您认为最恰当的分类**

**** 定义 ****

**a）理想自我：**人们自己以及他们的父母和朋友希望他们拥有的性格、思想和信念。
**b）应该自我：**人们自己以及他们的父母和朋友认为他们有义务或责任应该具备的性格、思想和信念。
**c）正面的真实自我：**人们内心深处真实的积极向上的性格、思想和信念。
**d）负面的真实自我：**人们内心深处真实的消极负面的性格、思想和信念。

|  | **理想自我** | **应该自我** | **正面的真实自我** | **负面的真实自我** |
| --- | --- | --- | --- | --- |
| 诚实 |  |  |  |  |
| 做白日梦 |  |  |  |  |
| 谨慎 |  |  |  |  |
| 幽默 |  |  |  |  |
| 冲动 |  |  |  |  |
| 嚣张跋扈 |  |  |  |  |
| 有责任心 |  |  |  |  |
| 客观公正 |  |  |  |  |
| 贪婪 |  |  |  |  |
| 现实 |  |  |  |  |
| 肤浅 |  |  |  |  |
| 有礼貌 |  |  |  |  |
| 有恒心 |  |  |  |  |
| 孝顺 |  |  |  |  |
| 虚荣 |  |  |  |  |
| 有耐心 |  |  |  |  |
| 粗鲁 |  |  |  |  |
| 神经质 |  |  |  |  |
| 慷慨 |  |  |  |  |
| 自律 |  |  |  |  |
| 愤世嫉俗 |  |  |  |  |
| 独立 |  |  |  |  |
| 反社会 |  |  |  |  |
| 直率 |  |  |  |  |
| 谦虚 |  |  |  |  |
| 自信 |  |  |  |  |
| 自负 |  |  |  |  |
| 节俭 |  |  |  |  |
| 不文明 |  |  |  |  |
| 正直 |  |  |  |  |

1. **在匿名的网络世界中，您会选择遵循内心真实的性格、思想和信念（即“真实自我”）吗？**
2. **是什么原因促使您在网上表现出 “真实自我”（特别是负面的“真实自我”）呢？**
3. **您每天平均的上网时间大约是_____________小时。[例如：2.3小时]**
4. **您的性别？**

□男 □女

1. **您的年龄？**

- 20岁以下
- 20-24
- 25-29
- 30-34
- 35-39
- 40-44
- 45-49
- 50或50以上

1. **您已获取的最高学位是什么？**

- 高中或高中以下
- 职业教育/专科
- 本科
- 硕士
- 博士

1. **您有几年工作经验？ _____年**
2. **您目前的工作领域？**

- 在读
- 未就业
- 退休
- 管理
- 商务/金融
- 计算机/数学
- 建筑/工程
- 生命/自然/社会科学
- 社区服务
- 法律
- 教育
- 艺术
- 卫生保健
- 食品
- 保洁/维护
- 个人护理服务
- 销售
- 行政
- 农业/渔业
- 制造业
- 运输
- 其它______

***感谢您的参与！***
