# Supplementary material for: Disinhibition of negative true self for identity reconstructions in cyberspace: Advancing self-discrepancy theory for virtual setting
Source: PLoS One. 2017 Apr 11;12(4):e0175623. doi: 10.1371/journal.pone.0175623 (PMC5388501; doi:10.1371/journal.pone.0175623)
Supplement: S2 File — (DOCX) [file pone.0175623.s002.docx]

**The expression of true self online**

The emergence of Internet changes the way people interact with others and constitutes a unique opportunity for self-expression, making it possible to express the traits that are hidden in one’s heart. In the anonymous online world, individual is able to express their true self with less fear of sanctions and disapproval by others. **True self was defined to represent what people** **intrinsically think and believe.** This study will try to explore the difference of individuals’ self-expression in online and offline world, which will mainly focus on the expression of the true self.

Thank you for your participation! **The participation of this study is voluntary and anonymous. All the collected data will only be used in academic research, only summarized data will be presented.**

1. **Do you agree that** **people have both positive and negative personality, minds, beliefs and consciousness?**
2. **Compared to the real world, do you think that you** **can express your true self more freely and openly online?**
3. **Generally, whether the following personality traits are suitable to be expressed in PHYSICAL WORLD?**

|  | Yes | No |
| --- | --- | --- |
| Honest |  |  |
| Daydreamer |  |  |
| Cautious |  |  |
| Humorous |  |  |
| Impulsive |  |  |
| Domineering |  |  |
| Responsible |  |  |
| Objective |  |  |
| Greedy |  |  |
| Materialistic |  |  |
| Superficial |  |  |
| Polite |  |  |
| Persistent |  |  |
| Obedient |  |  |
| Vain |  |  |
| Patient |  |  |
| Rude |  |  |
| Neurotic |  |  |
| Generous |  |  |
| Disciplined |  |  |
| Cynical |  |  |
| Independent |  |  |
| Antisocial |  |  |
| Frank |  |  |
| Modest |  |  |
| Self-confident |  |  |
| Conceited |  |  |
| Thrifty |  |  |
| Uncivil |  |  |
| Righteous |  |  |

1. **Apart from the words mentioned above, please provide two or three personality traits that can be expressed online, but NOT SUITABLE to be expressed in real world.**
2. **Please classify the words into suitable categories based on the definitions and your understanding in general:**
3. **Ideal self:** represents the traits or characteristics that someone wishes or aspires to possess
4. **Ought self:** represents the traits or characteristics that someone believes himself/herself *has the obligation, duty, or responsibility to possess.*
5. **Positive true self:** *Positive personality, minds, beliefs and consciousness that people intrinsically think, believe and behave accordingly.*
6. **Negative true self****:** *Negative personality, minds, beliefs and consciousness that people intrinsically think, believe and behave accordingly.*

|  | **Ideal self** | **Ought self** | **Positive true self** | **Negative true self** |
| --- | --- | --- | --- | --- |
| Honest |  |  |  |  |
| Daydreamer |  |  |  |  |
| Cautious |  |  |  |  |
| Humorous |  |  |  |  |
| Impulsive |  |  |  |  |
| Domineering |  |  |  |  |
| Responsible |  |  |  |  |
| Objective |  |  |  |  |
| Greedy |  |  |  |  |
| Materialistic |  |  |  |  |
| Superficial |  |  |  |  |
| Polite |  |  |  |  |
| Persistent |  |  |  |  |
| Obedient |  |  |  |  |
| Vain |  |  |  |  |
| Patient |  |  |  |  |
| Rude |  |  |  |  |
| Neurotic |  |  |  |  |
| Generous |  |  |  |  |
| Disciplined |  |  |  |  |
| Cynical |  |  |  |  |
| Independent |  |  |  |  |
| Antisocial |  |  |  |  |
| Frank |  |  |  |  |
| Modest |  |  |  |  |
| Self-confident |  |  |  |  |
| Conceited |  |  |  |  |
| Thrifty |  |  |  |  |
| Uncivil |  |  |  |  |
| Righteous |  |  |  |  |

1. **In anonymous online environment,** **will you choose to behave according to your true self?**
2. **What motivates you to express your true self online (especially negative true self)?**
3. **On average, how much time do you spent online per day?**

Hour(s) [For example: 2.3 hours]

1. **What’s your gender?**

□ Male □ Female

1. **How old are you?**

- Under 20
- 20-24
- 25-29
- 30-34
- 35-39
- 40-44
- 45-49
- 50 or older

1. **What is the highest level of education you have completed?**

- High school or lower
- Vocational/Technical school
- Bachelor’s degree or equivalent
- Master’s degree or equivalent
- Doctorate degree

1. **How long is your working experience?**

_________Years

1. **Which of the following categories best describes your primarily area of employment?**

- Student
- Unemployed
- Retired
- Management
- Business / financial operations
- Computer / mathematical
- Architecture and engineering
- Life, physical, and social science
- Community and social services
- Legal occupations
- Education, training, and library
- [Arts](https://en.wikipedia.org/wiki/Category:Arts_occupations), [design](https://en.wikipedia.org/wiki/Category:Design_occupations), entertainment, [sports](https://en.wikipedia.org/wiki/Category:Sports_occupations_and_roles)
- Healthcare
- Food industry
- Cleaning and maintenance
- Personal care and service
- Sales
- Office and administrative support
- Farming, fishing, and forestry related
- Manufacturing
- Transportation and material moving
- Others_______________________

***Thank you for your participation!***
